# Follow-up analysis of lesion characteristics of enchondromas and atypical cartilaginous tumours of the knee and shoulder region on MRI

**DOI:** 10.1007/s00330-024-11106-7

**Published:** 2024-10-16

**Authors:** Johannes Nikolaus Woltsche, Maria Anna Smolle, Dieter Szolar, Andreas Leithner

**Affiliations:** 1 Department of Orthopaedics and Trauma, Medical University of Graz, Graz, Austria; 2 Diagnostikum Graz, Graz, Austria

**Keywords:** Cartilaginous tumours/atypical, Enchondroma, Imaging, Follow-up studies, Extremities

## Abstract

**Objective:**

Enchondromas (ECs) and atypical cartilaginous tumours (ACTs), respectively, represent benign and intermediate cartilaginous bone tumours. Differentiation between these tumour entities bears difficulties, as histology and MRI cannot always provide exact diagnoses. Observation of the natural course of ECs/ACTs via follow-up MRIs might support tumour distinction without needing biopsy harbouring sampling error.

**Materials and methods:**

Reports of patients that had undergone MRI exams of the knee (*n* = 44.762) or shoulder (*n* = 21.550) at a single radiology institute between 01.01.2007 and 01.03.2020 were searched for ECs/ACTs with at least one follow-up MRI. Scans of 176 patients (with 182 cartilage lesions) fulfilling these criteria were subsequently re-examined together with corresponding MRI reports to evaluate morphological tumour development over time, focusing on potential alterations of lesion size, tumour-related oedema, and scalloping.

**Results:**

Median follow-up time was 27 ± 53 months for knee tumours and 26 ± 32 months for shoulder lesions. Presence of tumour growth was significantly higher in ACTs than in ECs both at the knee (*p* = 0.04) and shoulder (*p* = 0.03). While ACTs were associated with median tumour growth rates of 0.039 mm/month (knee) and 0.083 mm/month (shoulder), ECs of the knee and shoulder showed lower median growth rates equivalent to 0.0 mm/month (*p* < 0.01, *p* < 0.01). ECs and ACTs both presented stable regarding tumour-related oedema and scalloping during follow-up.

**Conclusion:**

ACTs and ECs show different tumour growth rates. Growth rates are slow for both, ECs and ACTs, supporting the current concept of watchful waiting. ECs may decrease in size. Follow-up MRIs may support the radiological differentiation of cartilage lesions.

**Key Points:**

***Question*** *Both singular MRI and histological examination have limitations regarding differentiation of enchondromas (EC) and atypical cartilaginous tumours (ACTs).*

***Findings*** *Median ACT growth rates were 0.039* *mm/month (knee) and 0.083* *mm/month (shoulder), while median growth rates of EC in the knee and shoulder were 0.0* *mm/month.*

***Clinical relevance*** *Active surveillance is a safe strategy when dealing with ECs and ACTs of the long bones; follow-up MRIs may support tumour distinction of cartilage lesions, as ECs and ACTs show different growth behaviour.*

## Introduction

Enchondromas (EC) are benign primary bone tumours that occur within the medullary cavity and consist of tumour cells producing a cartilaginous matrix [1]. Its malignant counterpart constitutes chondrosarcoma (CS), which can be subdivided into three grades: Grade 1 (G1) and grade 2 (G2) CSs comprise the majority of malignant cartilage tumours and usually show indolent clinical behaviour as well as low potential of metastasis, whereas grade 3 (G3) CS is associated with a higher likelihood to metastasise [2]. In 2020, a new classification for cartilage tumours of bone was introduced by the WHO: G1 CS of the appendicular skeleton was considered a separate tumour entity termed “atypical cartilaginous tumour” (ACT). This change in terminology should highlight that ACTs do not metastasise and show less aggressive local behaviour than G1 CSs of the axial skeleton [3].

As opposed to ACTs, which are often referred to as intermediate tumours, ECs represent truly benign lesions that are not associated with locally destructive growth [3]. Therefore, treatment strategies differ between ACTs and ECs, with intralesional resection (curettage and local adjuvant therapy) or active surveillance being recommended for intermediate cartilage tumours [4], whereas benign lesions do not require surgery as long as they remain asymptomatic [5].

Due to these differences regarding treatment strategies, the distinction between ACT and EC is an essential diagnostic task. However, identification of cartilage lesions’ dignity is considered difficult, as ECs and ACTs present with numerous morphologic similarities on MRI [6]. Even histopathological examination—considered the diagnostic gold standard—cannot always reliably differentiate between benign and intermediate lesions due to potential sampling error [4, 7]. Furthermore, biopsy is associated with a risk of tumour cell contamination of the biopsy tract, especially worrisome when dealing with high-grade CSs [4].

Therefore, many research groups have attempted to define new imaging criteria on MRI that support radiological differentiation between ECs and ACTs [8–10]. These criteria, in combination with rapidly improving imaging resolution, have contributed to the raised sensitivity of MRI over the last years. With histological examination bearing its disadvantages, MRI-based tumour differentiation of cartilage lesions is nowadays indispensable [4].

Besides the identification of differential tumour criteria on singular imaging scans, focus was also laid on the development/natural course of cartilaginous tumours during follow-up (FU): All studies on this subject showed that most benign/intermediate cartilage tumours remain stable and do not progress with time [11–14]. Therefore, active surveillance was proposed as a safe strategy for these intraosseous cartilaginous lesions: Sampath et al [11] recommended FU MRI 12 months after the initial diagnosis of EC/ACT, and Deckers et al suggested that patients should undergo their first FU MRI after 6 months [14].

However, despite evaluating the natural course of cartilage lesions, these studies did not differentiate between ECs and ACTs [11–14]. Therefore, it has not yet been clarified whether ECs and ACTs show differential morphological development over time, which could be another criterium supporting the radiological distinction of these tumour entities. To the best of our knowledge, the current study is the first one to investigate this issue. Furthermore, this is the largest-sized study so far to examine the natural course of ECs and ACTs of the long bones.

## Materials and methods

### Study population

This study has been approved by the local ethics committee (No. 36-070 ex 23/24). It was based on a retrospective analysis of 44.762 patients with an MRI of the knee and 21.550 patients with an MRI of the shoulder taken between 01.01.2007 and 01.03.2020 at a single radiology centre. Further details on the patient cohorts have been published previously [15, 16].

**Fig. 1:**
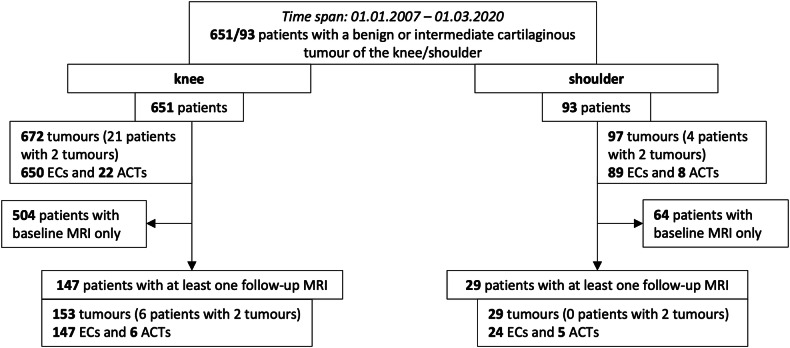
Flow-chart depicting the filtering of patients with an EC/ACT of the knee or shoulder, who received FU-MRIs

As depicted in Fig. 1, 504 patients with cartilage tumours of the knee and 64 patients with EC/ACT of the shoulder had to be excluded from the current study, as they had only received one MRI (baseline MRI) throughout the study period. The remaining 176 patients (147 patients with EC/ACT of the knee and 29 patients with EC/ACT of the shoulder) had undergone baseline MRI and at least one follow-up MRI. As 6 of these 176 patients had two cartilaginous tumours at the same time, 182 lesions in total (171 ECs, 11 ACTs) could be finally included. Patients had been referred to the radiology institute for different reasons: 38 patients (21.6%) had received MRI due to suspected/known cartilage lesion, 97 patients (55.1%) due to other reasons, and indication of MRI had not been documented in 41 cases (23.3%).

### Study design

In a first step, baseline and follow-up MRI reports (written by expert radiologists) of the included 176 patients were analysed by one of the co-authors, who had undergone extensive training regarding image interpretation of benign and intermediate cartilaginous tumours. All reports were re-examined together with the corresponding MRI scans and then filtered for the following data: patient gender and age, date and number of MRIs performed, tumour size, periosteal reaction, medullary oedema, endosteal scalloping, affected bone, tumour location within the affected bone, and location in relation to medullary canal.

Second, an expert orthopaedic oncologist re-examined all baseline and follow-up MRIs of the eleven included ACTs, again focusing on the above-mentioned tumour characteristics. All the findings of the radiologists were re-evaluated by this orthopaedic oncologist, who reached similar results.

### Lesion analysis

Intraosseous tumours presenting as smooth or lobulated lesions with tiny calcifications and showing high signal intensity on PD-FS-weighted (proton density, fat-suppressed) images and low signal intensity on PD-weighted as well as T1-weighted images were referred to as cartilage lesions.

Radiological differentiation between ECs and ACTs was performed based on the following criteria: Lesion size over 4.9 cm, deep endosteal scalloping (involvement of more than 2/3 of the bone cortex), tumour-related medullary oedema, and periosteal reaction. These features are highly indicative of aggressive tumour behaviour [4, 8, 9, 17]. Therefore, all lesions positive for at least one of these features were classified as ACTs.

Determination of tumour size was performed via measurement of the maximum lesion diameter in the coronal view. For size measurement, anatomical structures adjacent to the lesions were utilised as supporting landmarks to visualise the tumour in the same sectional plane across baseline and all follow-up MRIs.

**Fig. 2:**
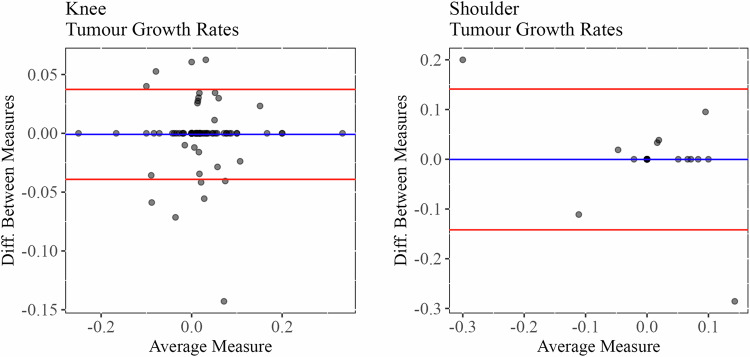
Comparison of tumour growth rates of knee and shoulder as calculated on the basis of measurements 1 and 2. For calculation of the difference between measures, measurement 2 was subtracted from measurement 1

To evaluate the accuracy and reproducibility of size determination, measurements that had been carried out at primary time point of data acquisition (measurement 1) were repeated one year thereafter (measurement 2) by an extensively trained co-author. Estimation of intraindividual reproducibility regarding tumour size evaluation at baseline and last follow-up was performed via calculation of coefficients of variation (ratio of standard deviation of measurement 1 and 2 to the mean of measurement 1 and 2 followed by multiplication with 100%). Comparison of measurements 1 and 2 for cartilage tumours of the knee revealed mean coefficients of variation of 1.8% (lesion size at baseline) and 1.8% (lesion size at last follow-up), whereas for shoulder tumours, mean coefficients of variation were 1.7% (lesion size at baseline) and 2.0% (lesion size at last follow-up). Furthermore, a comparison of tumour growth rates based on measurements 1 and 2 was performed via Bland-Altman plots as depicted by Fig. 2. As all of these evaluations indicated high reproducibility of tumour size measurement, changes in lesion size of at least 1 mm were referred to as statistically significant.

### Histology

After having completed radiological and clinical data acquisition, all patients with radiologically diagnosed ACTs (*n* = 11) were searched for in the hospital’s medical documentation system to investigate whether these patients had undergone tumour surgery/biopsy with histological tumour evaluation. However, only one of eleven patients had undergone tumour surgery and histopathology confirmed the radiological diagnosis of an ACT.

#### MRI

Patients received MRI examinations via 3-T MRI systems with a 15/18 channel coil for knee and a 16 channel coil for shoulder (Siemens Magnetom Skyra/Siemens Magnetom Vida). Detailed descriptions of all sequences acquired have been published previously [15, 16].

### Statistical analysis

All statistical analyses were performed with R version 4.2.1 (© 2022 The R Foundation for Statistical Computing). Normally distributed variables are represented as means with corresponding standard deviations, and non-normally distributed variables are given as medians with corresponding interquartile ranges (IQR). Fisher’s exact test was applied for the assessment of statistical differences in ordinary variables and *t*-test/Wilcoxon rank sum test was used for normally distributed/non-normally distributed metric variables. A *p*-value < 0.05 was considered statistically significant.

The following parameters were calculated for each lesion based on the data obtained: follow-up time, alteration of tumour size, tumour growth, tumour growth rate, alteration of periosteal reaction, alteration of medullary oedema and alteration of endosteal scalloping.

## Results

### Cartilage tumours of the knee

The included 153 cartilaginous lesions of the knee (147 ECs, 6 ACTs) had a median follow-up time (time difference between baseline MRI and last follow-up MRI) of 27 ± 53 months with the majority of tumours receiving baseline MRI and one follow-up MRI (Table 1). Mean age of all patients included (*n* = 147) was 49.8 ± 12.7 years and 52.4% (*n* = 77) of them were female. There were no significant differences between patients with EC and ACT regarding gender or age.

**Table 1:** Characteristics of ECs and ACTs of the knee and their development during FU

	Total count (*n* = 153)	ECs (*n* = 147)	ACTs (*n* = 6)	*p*-value
FU time (months) median ± IQR	27 ± 53	27 ± 54	31 ± 41	0.58*
Number of received MRIs median ± IQR	2 ± 1	2 ± 1	4.5 ± 2.5	0.01*
Tumour size BL (mm) median ± IQR	15.0 ± 13.0	15.0 ± 12.0	41.0 ± 26.3	< 0.01*
Tumour size last FU (mm) median ± IQR	15.0 ± 13.0	15.0 ± 12.0	43.5 ± 25.5	< 0.01*
Tumour size development				0.04**
Progression	48 (31.4%)	43 (29.3%)	5 (83.3%)	
Stable	87 (56.8%)	86 (58.5%)	1 (16.7%)	
Regression	18 (11.8%)	18 (12.2%)	0 (0.0%)	
Tumour growth (mm) median ± IQR	0.0 ± 1.0	0.0 ± 1.0	1.5 ± 1.8	< 0.01*
Growth rate (mm/month) median ± IQR	0 ± 0.017	0 ± 0.012	0.039 ± 0.024	< 0.01*
Bone				0.12**
Femur (distal)	110 (71.9%)	106 (72.1%)	4 (66.7%)	
Tibia (proximal)	28 (18.3%)	28 (19.0%)	0 (0.0%)	
Fibula (proximal)	14 (9.2%)	12 (8.2%)	2 (33.3%)	
Patella	1 (0.6%)	1 (0.7%)	0 (0.0%)	
Location				< 0.05**
Epiphysis	16 (10.5%)	16 (10.9%)	0 (0.0%)	
Epimetaphysis	20 (13.1%)	18 (12.3%)	2 (33.3%)	
Metaphysis	90 (58.8%)	89 (60.5%)	1 (16.7%)	
Metadiaphysis	10 (6.5%)	9 (6.1%)	1 (16.7%)	
Diaphysis	16 (10.5%)	14 (9.5%)	2 (33.3%)	
Patella	1 (0.6%)	1 (0.7%)	0 (0.0%)	
Location in relation to medullary canal				0.40**
Central	64 (41.8%)	62 (42.2%)	0 (0.0%)	
Peripheral	89 (58.2%)	85 (57.8%)	6 (100.0%)	
Periosteal reaction BL				
Yes	0 (0.0%)	0 (0.0%)	0 (0.0%)	
No	153 (100.0%)	147 (100.0%)	6 (100.0%)	
Alteration of periosteal reaction during FU				
Yes	0 (0.0%)	0 (0.0%)	0 (0.0%)	
No	153 (100.0%)	147 (100.0%)	6 (100.0%)	
Medullary oedema BL				
Yes	0 (0.0%)	0 (0.0%)	0 (0.0%)	
No	153 (100.0%)	147 (100.0%)	6 (100.0%)	
Alteration of medullary oedema during FU				
Yes	0 (0.0%)	0 (0.0%)	0 (0.0%)	
No	153 (100.0%)	147 (100.0%)	6 (100.0%)	
Endosteal scalloping BL				< 0.01**
Superficial	2 (1.3%)	1 (0.7%)	1 (16.7%)	
Deep	4 (2.6%)	0 (0.0%)	4 (66.6%)	
No	147 (96.1%)	146 (99.3%)	1 (16.7%)	
Alteration of endosteal scalloping during FU				
Yes	0 (0.0%)	0 (0.0%)	0 (0.0%)	
No	153 (100.0%)	147 (100.0%)	6 (100.0%)	

* Wilcoxon rank sum test

** Fisher’s exact test

*p*-values highlighted in bold indicate statistical significance

**Fig. 3:**
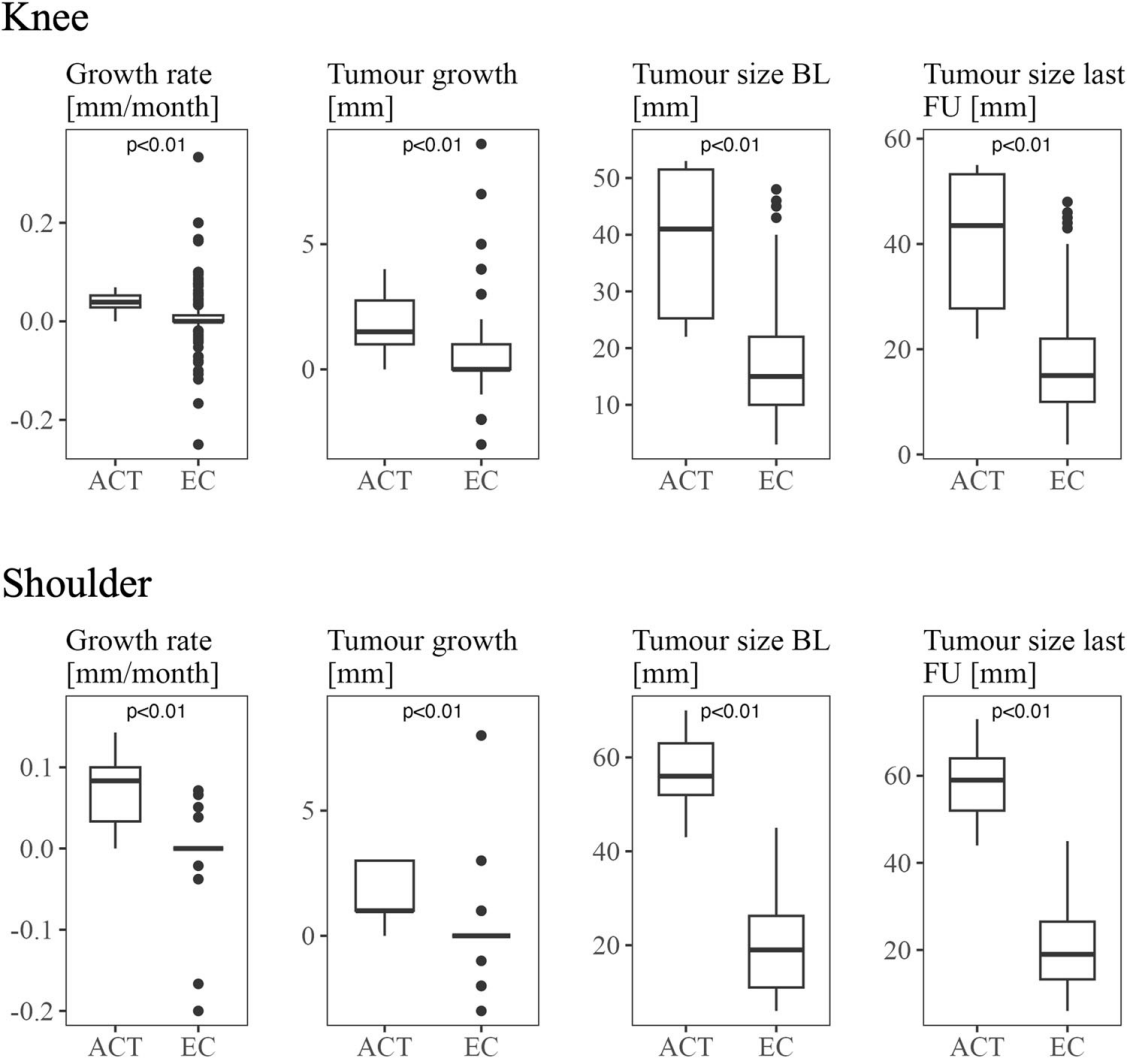
Boxplots depicting lesion size-related parameters of ACTs compared to ECs

While most ECs presented with stable lesion size during follow-up, 12.2% of ECs showed a decrease, and 29.3% showed an increase. On the other hand, 83.3% of ACTs presented with an increase in tumour size and no ACT with a decrease in lesion size. ACTs showed significantly higher growth rates than ECs. As depicted in Fig. 3, significant differences were found between ACTs and ECs regarding all lesion size-related parameters (tumour size at baseline MRI, tumour size at last follow-up MRI, tumour growth, growth rate).

Other morphologic tumour characteristics, such as periosteal reaction and medullary oedema, were neither revealed by any baseline MRI in the ACT or the EC group, nor did a single follow-up MRI show an alteration on this parameter. At baseline, deep endosteal scalloping was present in 66.6% of ACTs. Superficial scalloping was found in 16.7% of ACTs and 0.7% of ECs. However, both deep and superficial scalloping presented stable throughout the whole follow-up period for ACTs and ECs.

Table 2 depicts tumour characteristics for every ACT (*n* = 6) in detail. Follow-up MRIs of three ACTs revealed alterations in tumour morphology regarding lobulation, cartilage, calcification and configuration of extra-osseous parts of the lesion.

**Table 2:** Detailed description of characteristics of all ACTs of the knee (*n* = 6)

	ACT_K1	ACT_K2	ACT_K3	ACT_K4*	ACT_K5**	ACT_K6***
Number of received MRIs	2	2	4	5	5	5
Histology	No biopsy	ACT	No biopsy	No biopsy	No biopsy	No biopsy
FU time (months)	29	6	21	74	33	109
Tumour size BL (mm)	53	22	35	47	53	22
Tumour size last FU (mm)	55	22	36	51	54	25
Growth rate (mm/month)	0.069	0.000	0.048	0.054	0.030	0.028
Bone	Femur	Fibula	Femur	Femur	Femur	Fibula
Location	Diaphysis	Epimetaphysis	Metadiaphysis	Diaphysis	Metaphysis	Epimetaphysis
Location in relation to medullary canal	Peripheral	Peripheral	Peripheral	Peripheral	Peripheral	Peripheral
Endosteal scalloping BL	No	Deep	Deep	Deep	Superficial	Deep

* ACT_K4: increase of lobulation peripherally

** ACT_K5: increase of cartilage peripherally and increase of calcifications centrally

*** ACT_K6: increase of the extra-osseous part of the lesion

### Cartilage tumours of the shoulder

Age (EC: 52.9 ± 11.7 years; ACT: 62.6 ± 14.9 years (*p* = 0.15)) and gender (EC: 45.8% female; ACT: 40.0% female (*p* > 0.99)) were comparable between EC and ACT patients. As depicted in Table 3, median follow-up time for all cartilage lesions was 26 ± 32 months, and most patients had received three MRIs.

**Table 3:** Characteristics of ECs and ACTs of the shoulder and their development during FU

	Total count (*n* = 29)	ECs (*n* = 24)	ACTs (*n* = 5)	*p*-value
FU time (months) median ± IQR	26 ± 32	25 ± 35.75	30 ± 9	0.75*
Number of received MRIs median ± IQR	3 ± 2	3 ± 2.25	2 ± 1	0.25*
Tumour size BL (mm) median ± IQR	23.0 ± 27.0	19.0 ± 15.3	56.0 ± 11.0	< 0.01*
Tumour size last FU (mm) median ± IQR	24.0 ± 27.0	19.0 ± 13.3	59.0 ± 12.0	< 0.01*
Tumour size development				0.03**
Progression	8 (27.6%)	4 (16.7%)	4 (80.0%)	
Stable	17 (58.6%)	16 (66.6%)	1 (20.0%)	
Regression	4 (13.8%)	4 (16.7%)	0 (0.0%)	
Tumour growth (mm) median ± IQR	0.0 ± 1.0	0.0 ± 0.0	1.0 ± 2.0	< 0.01*
Growth rate (mm/month) median ± IQR	0 ± 0.033	0 ± 0.0	0.083 ± 0.067	< 0.01*
Bone				
Humerus	29 (100.0%)	24 (100.0%)	5 (100.0%)	
Scapula	0 (0.0%)	0 (0.0%)	0 (0.0%)	
Location				0.14**
Epiphysis	1 (3.4%)	1 (4.2%)	0 (0.0%)	
Epimetaphysis	2 (6.9%)	2 (8.3%)	0 (0.0%)	
Metaphysis	20 (69.1%)	18 (75.0%)	2 (40.0%)	
Metadiaphysis	5 (17.2%)	3 (12.5%)	2 (40.0%)	
Diaphysis	1 (3.4%)	0 (0.0%)	1 (20.0%)	
Scapula	0 (0.0%)	0 (0.0%)	0 (0.0%)	
Location in relation to medullary canal				0.13**
Central	10 (34.5%)	10 (41.7%)	0 (0.0%)	
Peripheral	19 (65.5%)	14 (58.3%)	5 (100.0%)	
Periosteal reaction BL				
Yes	0 (0.0%)	0 (0.0%)	0 (0.0%)	
No	29 (100.0%)	24 (100.0%)	5 (100.0%)	
Alteration of periosteal reaction during FU				
Yes	0 (0.0%)	0 (0.0%)	0 (0.0%)	
No	29 (100.0%)	24 (100.0%)	5 (100.0%)	
Medullary oedema BL				0.17**
Yes	1 (3.4%)	0 (0.0%)	1 (20.0%)	
No	28 (96.6%)	24 (100.0%)	4 (80.0%)	
Alteration of medullary oedema during FU				
Yes	0 (0.0%)	0 (0.0%)	0 (0.0%)	
No	29 (100.0%)	24 (100.0%)	5 (100.0%)	
Endosteal scalloping BL				< 0.01**
Superficial	1 (3.4%)	0 (0.0%)	1 (20.0%)	
Deep	2 (6.9%)	0 (0.0%)	2 (40.0%)	
No	26 (89.7%)	24 (100.0%)	2 (40.0%)	
Alteration of endosteal scalloping during FU				
Yes	0 (0.0%)	0 (0.0%)	0 (0.0%)	
No	29 (100.0%)	24 (100.0%)	5 (100.0%)	

* Wilcoxon rank sum test

** Fisher’s exact test

*p*-values highlighted in bold indicate statistical significance

In 80% of cases, ACTs showed an increase in tumour size during follow-up, compared to 16.7% of ECs. While 16.7% of shoulder ECs presented with regressing tumour size during follow-up, none of the ACTs showed a decreasing tumour size. The growth rate was significantly different between the groups. Furthermore, ACTs presented with higher values regarding tumour size at baseline and last follow-up as well as overall tumour growth (Fig. 3).

No tumour showed periosteal reaction, whilst one ACT presented with tumour-related medullary oedema at baseline, and three ACTs showed endosteal scalloping (superficial scalloping in one ACT, deep scalloping in two ACTs). All of these morphologic parameters presented stable throughout the follow-up period. Table 4 offers a detailed description of all shoulder ACTs and their tumour characteristics.

**Table 4:** Detailed description of characteristics of all ACTs of the shoulder (*n* = 5)

	ACT_S1	ACT_S2	ACT_S3	ACT_S4	ACT_S5
Number of received MRIs	2	2	2	3	4
Histology	No biopsy	No biopsy	No biopsy	No biopsy	No biopsy
FU time (months)	45	30	12	30	21
Tumour size BL (mm)	52	70	63	43	56
Tumour size last FU (mm)	52	73	64	44	59
Growth rate (mm/month)	0.000	0.100	0.083	0.033	0.143
Bone	Humerus	Humerus	Humerus	Humerus	Humerus
Location	Metadiaphysis	Diaphysis	Metaphysis	Metadiaphysis	Metaphysis
Location in relation to medullary canal	Peripheral	Peripheral	Peripheral	Peripheral	Peripheral
Endosteal scalloping BL	Deep	No	Superficial	Deep	No
Medullary oedema BL	Yes	No	No	No	No

### ACTs of the knee and shoulder

**Fig. 4:**
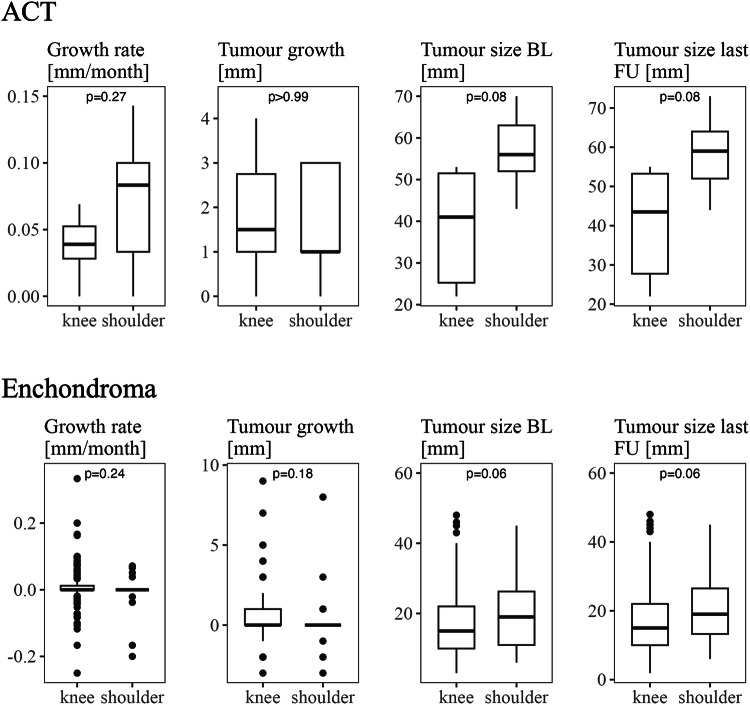
Boxplots depicting lesion size-related parameters of cartilage tumours of the knee compared to EC/ACTs of the shoulder

Comparison of ACTs (Fig. 4) of different sites (knee and shoulder) revealed no difference between the groups regarding tumour growth rate and morphologic characteristics like periosteal reaction, medullary oedema or endosteal scalloping. Tumour size at baseline and last follow-up showed higher values for ACTs of the shoulder than the knee, though these results were not statistically significant.

### Enchondromas of the knee and shoulder

Like ACTs, ECs around the knee neither showed a different growth rate compared to ECs of the shoulder, nor was there a site-specific variation with regards to superficial endosteal scalloping. ECs of the shoulder tended to present with higher lesion size at baseline and last follow-up than ECs of the knee (Fig. 4).

## Discussion

In need of further radiological criteria to differentiate between benign and intermediate cartilage tumours, this study was performed to investigate whether ECs and ACTs of the long bones showed differential morphological development on MRI over time. We studied the natural course of 182 cartilaginous lesions (171 ECs, 11 ACTs) of the knee and shoulder. While both ECs and ACTs presented as stable regarding the status of periosteal reaction, scalloping, and tumour-related oedema, significant differences were discovered for lesion size-related parameters: ACTs of the knee and shoulder showed significantly higher tumour growth rates than ECs. Furthermore, no ACT showed a decrease in tumour size over time, whereas 12.2% and 16.7% of ECs at the knee and shoulder, respectively, demonstrated tumour regression.

The natural course of cartilage lesions has been studied before [11–14], however, these studies did not radiologically differentiate between ECs and ACTs when evaluating tumour development over a certain time span. Kumar et al [11] analysed cartilage lesions of the knee only. The authors of the other three studies [12–14] examined lesions of knee and shoulder, however, they did not provide separate analysis depending on tumour site. Furthermore, the current study is based on a larger cohort of cartilage tumours (*n* = 182) than those four studies [11–14]. In line with the findings of the studies performed by Chung et al [13] and Deckers et al [14], this analysis confirms that spontaneous regression of cartilaginous lesions at the knee and shoulder is possible. Notably, the definition of tumour regression differs from study to study--when focusing on the development of tumour size only, Deckers et al [14] reported a decrease in lesion size in 13.3% of 128 cartilaginous tumours; this is comparable to the percentages revealed within this study (12.2% (knee); 16.7% (shoulder)). Chung et al [13], however, found that 52.4% of 21 ECs/ACTs were associated with spontaneous regression in tumour size. The study of Deckers et al [14] and the current study were based on larger sample sizes than the study by Chung et al [13]; therefore, these numbers might better reflect the true percentage of cartilaginous lesions that regress.

**Fig. 5:**
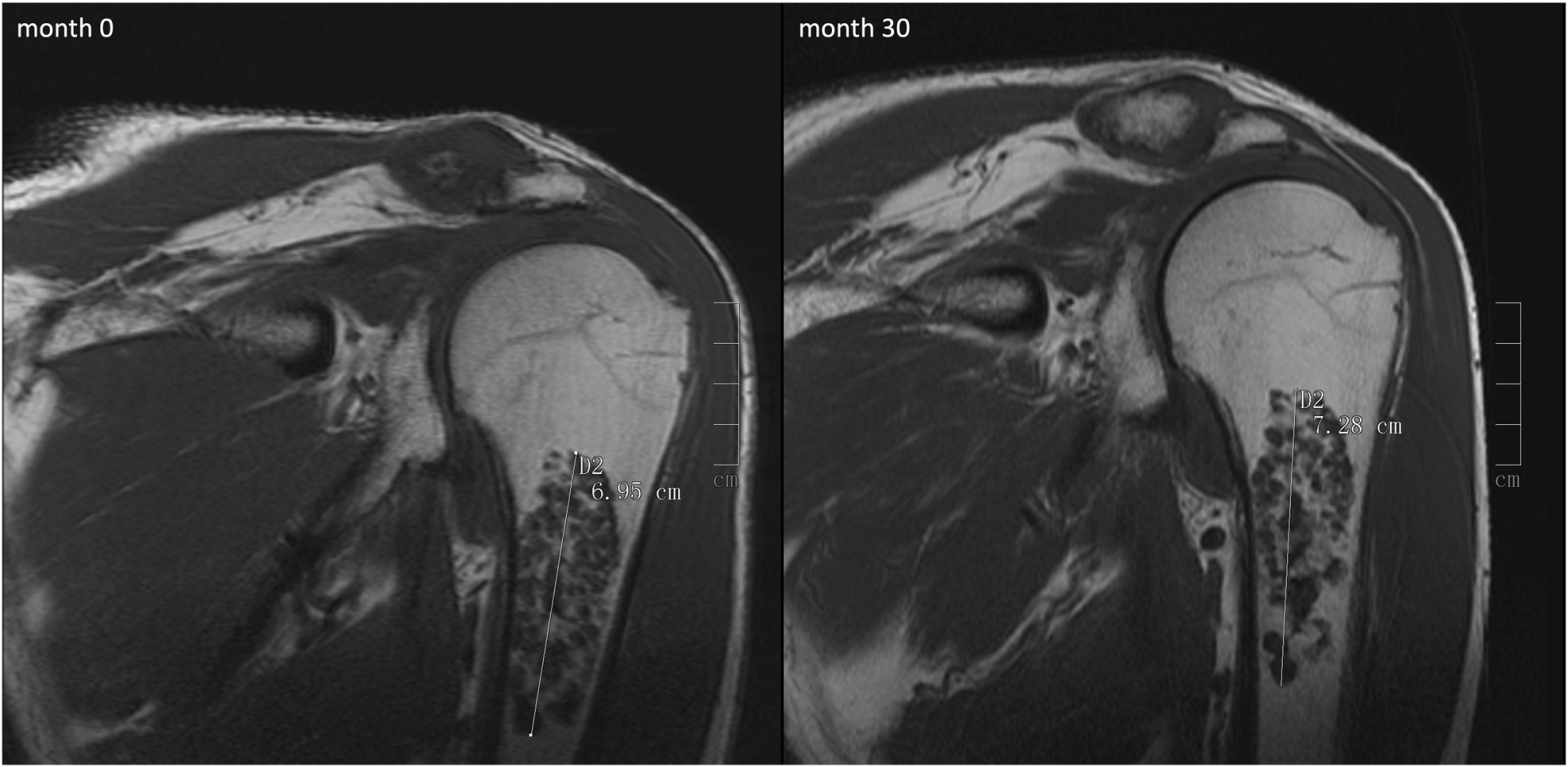
ACT_S2 showed an increase of 3 mm in cranio-caudal tumour extension within 30 months of follow-up (coronal T1 spin echo sequences)

The mentioned follow-up studies have shown that 13.0 to 23.9% of cartilage lesions of the appendicular skeleton present with increasing tumour size [11–14]. Results of the current study reveal that a slightly higher percentage of cartilage lesions show growth during follow-up. While 31.4% of cartilaginous neoplasms around the knee joint were associated with an increase in size, 27.6% of shoulder tumours showed growth. These slightly higher percentages might be explained by the fact that Kumar et al [11] and Deckers et al [14] classified lesions as progressive when presenting with tumour growth of at least 6 mm [11]/3 mm [14], whereas an increase in size of 1 mm was interpreted as growth in the current study. As pointed out previously, our data suggest that increasing tumour size occurs with a significant higher frequency in ACTs than ECs (Fig. 5). This characteristic is not specific for ACTs, however, as 29.3% of knee ECs and 16.7% of shoulder ECs also present with increasing tumour size. Radiological distinction towards tumour dignity was not performed in any of the studies discussed above [11–14]. However, when taking together all lesions of the four studies [11–14] that underwent biopsy/surgery due to tumour growth with histopathology available (*n* = 10), seven of these lesions were diagnosed as ACT and two as EC (histology of one tumour was inconclusive due to sample error [14]). This might support the finding of the current study that ACTs are associated with tumour growth at a higher frequency than ECs.

Comparison of the parameter tumour growth within and in between studies can be biased due to differences in length of follow-up periods. To avoid this bias, the current study also focused on the analysis of tumour growth rates. As Chung et al [13] and Deckers et al [14] published data on the extent of tumour growth and FU time of progressive cartilaginous lesions, growth rates of these lesions could be calculated (ratio of median tumour growth (mm) and median follow-up time (month)). As depicted in Table 5, cartilage lesions with an increase in tumour size showed a growth rate of 0.109 mm/month in the study of Chung et al [13] and 0.106 mm/month in the study of Deckers et al [14], whereas in the current study, progressive lesions presented with slower growth (0.018 mm/month). As opposed to the current study, Deckers et al [14] did not classify lesions as progressive if they showed tumour growth of less than 3 mm. This may serve as an explanation for the higher median tumour growth rate reported in that study [14] compared to our findings. Furthermore, Deckers et al [14] recruited all patients at a tertiary care centre, with 73% of included patients had received radiological examinations due to already known cartilage lesions, whereas all patient data of the current study were collected at a private radiologic institute, and only 21.6% of patients had undergone MRI because of suspected cartilaginous tumour. Therefore, the data of Deckers et al [14] might be based on a cohort of more complex cartilage tumours. However, these lesions do not represent the majority of cartilage lesions, as most ECs present asymptomatically and are most frequently discovered as incidental findings via imaging performed for other reasons [5, 7]. Bearing these issues in mind, the true average tumour growth rate of progressive cartilage lesions may be more accurately reflected by the data of the current study.

**Table 5:** Comparison of cartilage tumour growth rates of knee and shoulder of all studies that contain data on tumour growth and follow-up time

	Total number of lesions examined (*n*)	Number of growing lesions (*n*, %)	Median follow-up time (in months, range)	Median tumour growth (in mm, range)	Calculated tumour growth rate (in mm/month)
Current study	182	56 (30.8%)	53 (3–158)	1 (1–9)	0.018
Chung et al [13]	21	5 (23.8%)	51 (36–56)	5.6 (0–15)	0.109
Deckers et al [14]	128	17 (13.3%)	47 (30–138)	5 (3–23)	0.106

The “calculated tumour growth rate” corresponds to the ratio of “median tumour growth” and “median follow-up time”

The results of this study should be interpreted with caution, as differentiation between ECs and ACTs was MRI-based only in most cases. Histopathological assessment is still considered the gold standard for distinction between benign and intermediate cartilaginous lesions, however, there is increasing evidence that MRI-based diagnoses are very accurate, as this method is not biased by potential sampling errors [4]. Therefore, it can be assumed that additional histopathological evaluation of all tumours would not have considerably changed the results obtained. This study is also limited by its retrospective design and heterogenous follow-up of lesions, which impairs drawing uniform solid conclusions on growth rates.

In summary, the current study revealed that ACTs of the knee and shoulder region are significantly more frequently associated with tumour growth than ECs, which may also decrease in size. ACTs and ECs show different tumour growth rates. Growth rates are slow for both, ECs and ACTs, supporting the current concept of watchful waiting. Benign and intermediate cartilage lesions present stable during follow-up regarding tumour-related oedema and scalloping. Follow-up MRIs may support the radiological differentiation of cartilage lesions.

## Abbreviations

ACT: Atypical cartilaginous tumour
BL: Baseline
CS: Chondrosarcoma
EC: Enchondroma
FU: Follow-up
G1, G2, G3: Grade 1, grade 2, grade 3
IQR: Interquartile range
PD: Proton density
